# Management of a nine litre abdominal wall seroma post ventral hernia repairs: A case report

**DOI:** 10.1016/j.ijscr.2020.06.034

**Published:** 2020-06-12

**Authors:** Danika Jurat, Parveen Kumar, Kim Goddard

**Affiliations:** General Surgery Department, Armadale Health Service, Australia

**Keywords:** Seroma, Mesh repair, Ventral wall hernia, Partial capsulectomy, Scarification, Case report

## Abstract

**Introduction:**

seromas are a common and challenging complication post mesh hernia repairs (Morales-Conde, 2012; Salamone, 2015; Vasilakis, 2014).

**Presentation of case:**

A 52-year-old male was referred to General Surgery with a large abdominal mass in the setting of morbid obesity and multiple abdominal operations culminating in a ventral hernia repair seven years prior. He had no other abdominal symptoms or malignancy red flags. Imaging demonstrated a large, complex anterior abdominal wall seroma of 40 × 25 × 20 cm. No seromas of this size and location have been reported in the literature.

**Discussion:**

Surgical management required drainage, partial capsulectomy and scarification of the remnants with multidisciplinary team involvement for recovery including general surgeons, anaesthesia, intensivists and allied health professionals.

**Conclusion:**

The patient recovered well from his operation.

## Highlights

• Seromas are common post hernia mesh repairs.
• Seromas present challenges to both patient and doctor.
• Drainage, partial capsulectomy & scarification is acceptable for resolution of large seromas with tissue preservation & defect prevention.

## 1 Introduction

Seromas are a challenging complication for both patient and doctor with fluid collection after a procedure that creates a dead space with disruption to lymphatics and vasculature structures [[1], [2], [3]]. Formation post mesh repair of a ventral hernia is a common complication with the incidence reported at variable rates ranging from 0.5 to 78% post laparoscopic repair and 30–50 % post open repair [1,2].

Acceptable management approaches for seromas include a conservative approach, percutaneous or surgical drainage. This case details a method of surgical drainage including fluid aspiration, partial capsulectomy and scarification.

This case report has been reported in line with SCARE criteria [4].

## 2 Patient information

A 52-year-old Caucasian male presented with a large anterior abdominal wall collection on a background of a mesh repair of a recurrent incisional hernia with multiple previous abdominal operations. The mass measured 40 × 25 × 20 cm on abdominal computed tomography in January 2020.

The patient’s initial surgery was a paraumbilical hernia repair in 2002 followed by exploration of the repair later that year following concerns of infection. The patient underwent further operative intervention for bowel obstruction requiring resection in 2003 and repair of an incarcerated ventral hernia in 2010. The patient received a penultimate, massive, recurrent incisional hernia repair with onlay 20 × 30 cm Ethicon physiomesh in 2013 at BMI 45. Subsequent to this he developed a vast collection requiring operative intervention in 2020. In the interim period his increasing abdominal girth was attributed to adiposity. In 2019, his general practitioner requested an abdominal computed tomography, identifying the collection and facilitating a referral to the General Surgery outpatient clinic.

On review in the General Surgery outpatient clinic the patient had massive truncal obesity, the vast majority of which, clinically and radiologically was secondary to the large collection. The patient’s comorbidities included morbid obesity, hypertension, high cholesterol, obstructive sleep apnoea, previous supraventricular tachycardia with ablation in 2013, diet controlled type two diabetes mellitus and was a previous smoker having quit in 2006. His medications included irbesartan, amlodipine, atorvastatin, amitriptyline, metformin, levothyroxine and diazepam nocte. He had no known drug allergies.

The patient was from home, normally independent with his activities of daily living and maintaining a job as an information technology (IT) professional.

## 3 Clinical findings

The patient was hemodynamically stable on presentation to the General Surgery outpatient clinic. He had massive truncal obesity resulting in a challenging abdominal examination. A mass of ill-defined margins was palpable on the right hand side. Level of fluctuance, margins and tethering were all difficult to establish with the patient’s body habitus.

## 4 Diagnostic assessment

The patient had repeat abdominal imaging over the course of his operations and subsequent complications. Relevant ones include:

An abdominal x-ray querying bowel obstruction was requested by the Emergency Department in 2010. This was post periumbilical hernia repair. This demonstrated ileus/subacute obstruction with recommendations to follow with a computed tomography scan of the abdomen. This was performed and demonstrated a recurrent ventral hernia with evidence to suggest incarceration with a loop of bowel herniating through and secondary partial bowel obstruction noted with dilatation of the proximal small bowel.

Three weeks post this ventral hernia repair, the patient represented with serous discharge superiorly and induration over the lower end of the wound. An ultrasound was performed for potential hernia recurrence or seroma collection. No evidence of recurrent hernia was demonstrated, multiple areas of deep subcutaneous fluid, possibly haematoma or seroma, were visualised. This was attributed to recent large mesh insertion and the patient was managed for a superficial skin infection on oral antibiotics in the setting of being clinically well and having only mildly elevated inflammatory markers. The discharge and induration resolved.

**Fig. 1:**
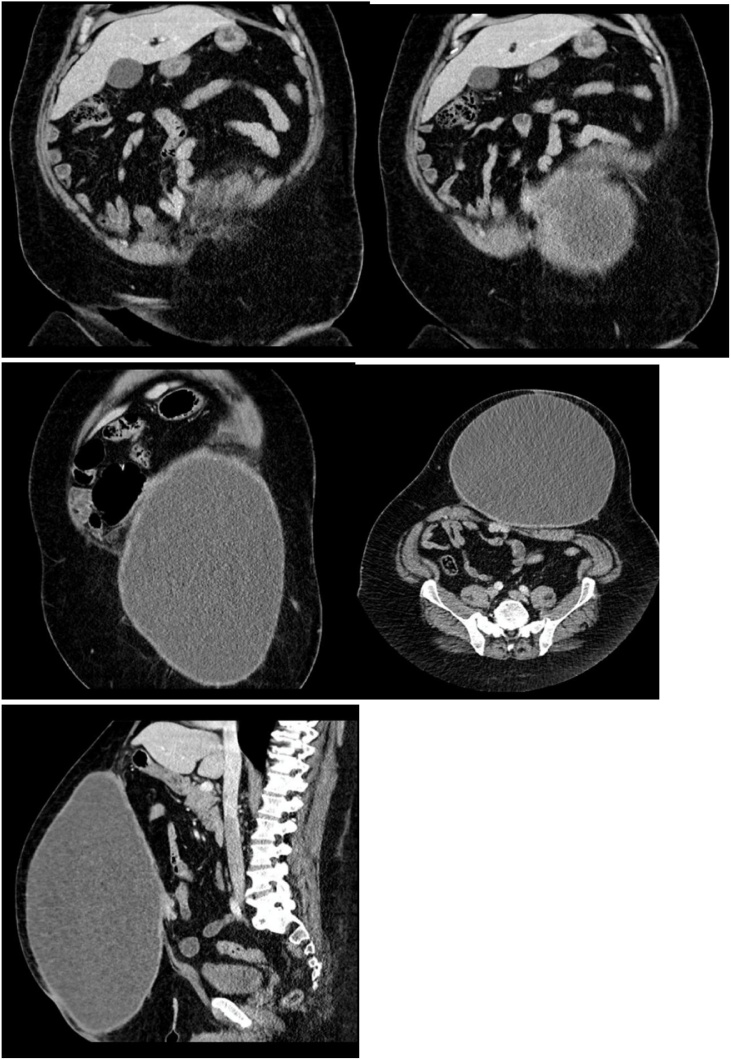
Pre-operative computed tomography imaging demonstrating seroma size.

A further abdominal computed tomography was performed in February 2013 demonstrating a further, massive ventral hernia. The patient underwent a repair, then repeat imaging in October 2013 for assessment of a post-operative collection. This demonstrated a collection of 67 × 31 × 70 mm cranial to the surgical sutures. The final pre-operative computed tomography of 2020 demonstrated a 40 × 25 × 20 cm collection [Fig. 1].

The patient’s pre-operative laboratory tests were unremarkable with normal inflammatory markers.

The most likely diagnosis for this patient was a seroma. Differentials for the mass included a large cyst or infected collection/abscess. The patient’s absence of systemic features such as fever and low inflammatory markers made an infected collection or abscess unlikely. Finally the patient’s multitude of seroma risk factors and impression intra-operative supported this diagnosis. Samples of fluid taken intra-operative returned nil bacterial growth on microscopy and culture. The patient’s histopathology sample of the fibrous capsule demonstrated no malignancy.

## 5 Therapeutic intervention

The patient was booked and consented for operative drainage/removal of the organised seroma with an intensive care unit bed reserved post-operative given the size of the undertaking and the patient’s comorbidities.

**Fig. 2:**
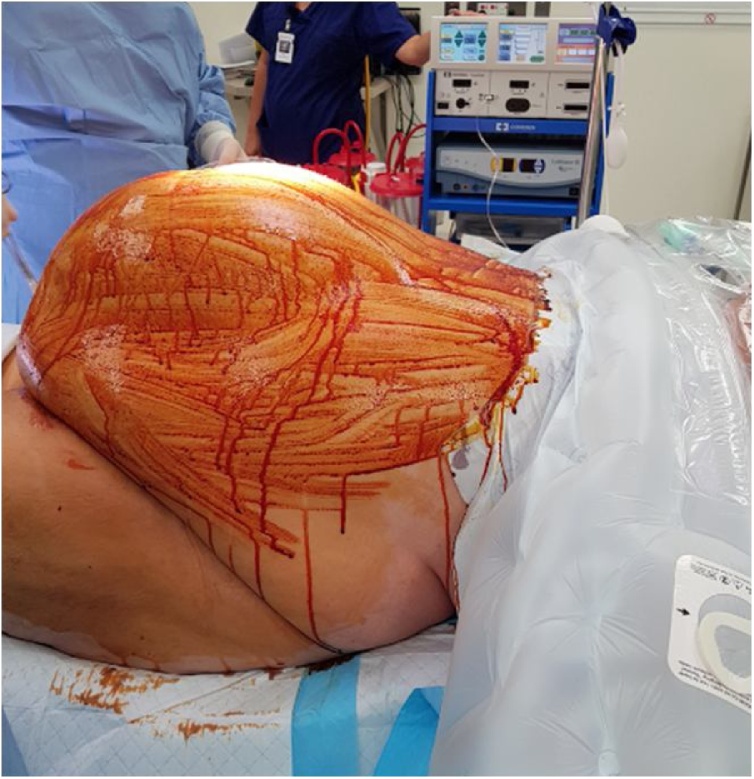
Immediately pre-operative photograph demonstrating seroma size.

**Fig. 3:**
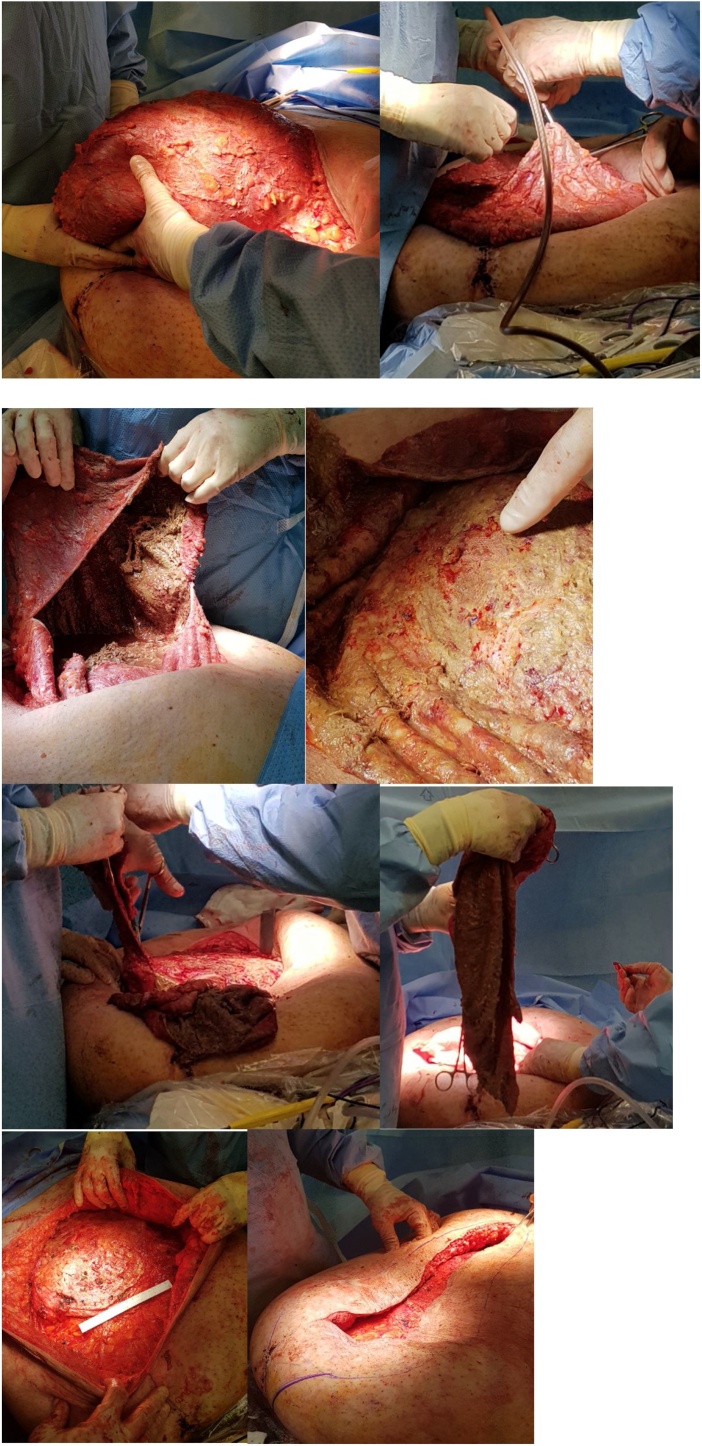
Intra-operative dissection, drainage, partial capsulectomy & demonstration of defect size.

The patient was positioned supine and administered a general anaesthetic. He was prepped with iodine and square draped [Fig. 2]. The operation was performed by a senior General Surgery consultant. Intra-operatively, post lateral dissection bilaterally down to sheath, the findings were of a large organised encapsulated seroma in the subcutaneous layer adherent to the anterior rectus sheath overlying mesh containing approximately 9.5 L of turbid fluid. The seroma was drained under controlled conditions and the thick walled sac was excised circumferentially down to sheath. The sheath was extensively curetted to remove thick slough and any visible mesh remnants excised. Tissel haemostatic agent was sprayed over the exposed floor of seroma and adherent sheath. The area was repeatedly washed with normal saline. The small recurrence at superior end was repaired with 1−0 nylon. An abdominoplasty was performed to remove redundant abdominal wall. Final closure was achieved with interrupted vicryl to dermis and staples to the skin. Two 19 French Blakes drains were inserted [Fig. 3]. The patient was admitted to the intensive care unit as planned post-operatively on clear fluids as tolerated. He continued on intravenous antibiotics with flowtrons and heparin 5000 units TDS for deep vein thrombosis prophylaxis.

## 6 Post-operative outcomes

The patient was admitted to ICU post-operative for monitoring in light of his comorbidities with an epidural in situ. This was removed day one post-operative and the patient was transferred to the General Surgery ward. He continued on intravenous cefazolin for seven days post-operative with two drains in situ requiring minimal regular and supplementary analgesia. The patient mobilised well daily both independently and with the physiotherapy team. He received mechanical and pharmacological deep vein thrombosis prophylaxis is the form of TED stockings and heparin subcutaneous injections. There was some concern surrounding mild erythema to the right side of the incision day three post-operative. The patient remained systemically well, afebrile, with nil discharge from or collection to the area. Nil bacteria was grown on swabs to the area and the Infectious Diseases team was consulted prophylactically. Given the patient’s improving inflammatory markers and lack of growth on swabs, he remained on cefazolin and the erythema resolved.

Clinically and biochemically the patient recovered well. He was discharged day seven post admission on a further five day course of oral antibiotics, with two drains in situ, daily community nursing review and weekly surgical doctor review. One drain was removed in the community when output was less than 30mls in 24 h.

On first review in the outpatient clinic the patient’s second drain had accidentally been preliminarily self-removed. Another drain was reinserted under ultrasound guidance. The patient was compliant with post-operative advice of no heavy lifting for 4–6 weeks.

## 7 Discussion

Seroma formation post mesh repair of a ventral hernia is a common complication with the incidence reported at variable rates up to 78 % post laparoscopic repair and 50 % post open repair [1,2]. No cases in the literature document one as extensive as this patient who was at high risk given his repeat, open operations, complications, mesh insertion and comorbidities including diabetes and morbid obesity.

Conservative management for this patient had incidentally been trialled for seven years with nil success. Percutaneous aspiration was a poor option given the complex nature of the collection, size, remaining dead space and capsule meaning almost certain re-collection. The risk of both percutaneous and surgical drainage of seromas is introduction of bacteria and creation of an infected collection. For improved patient outcome, optimum management is definitive treatment resulting in resolution of the collection with no subsequent infection or re-collection. Ideally the patient would have received a full capsulectomy but this was unattainable due to the size of the defect full resection would have left in the abdominal wall.

A case documented by Vasilakis et al. details a 4 year history development of a complex abdominal wall seroma [3]. Their patient was also post ventral herniorrhaphy with mesh, with the seroma measuring 10.1 × 17.3 × 17.3 cm on computer tomography. They were also unable to completely resect the seroma due to the anatomic relationship of the posterior aspect of the mesh and subsequently attached pseudocapsule. Their conclusion was that capsulectomy and scarification of the remnant pseudocapsule was an acceptable and safe surgical option for management of complex chronic abdominal wall seromas.

## 8 Conclusion

This multimodal approach of drainage, capsulectomy and scarification was utilised for this patient with approximately twice the size and duration of seroma. He will be closely monitored and followed up for potential recurrence or subsequent infection.

## Declaration of Competing Interest

The authors declare nil competing interests.

## Sources of funding for your research

The authors declare nil sources of funding.

## Ethical approval

The study is exempt from ethnical approval in my institution as a case report- it only requires patient consent which was obtained.

## Consent

Written informed consent was obtained from the patient for publication of this case report and accompanying images. A copy of the written consent is available for review by the Editor-in-Chief of this journal on request.

## Author contribution

Study concept & paper edits: Parveen Kumar.

Study concept & writing the paper: Danika Jurat.

Supervisor, study concept & paper edits: Kim Goddard.

## Guarantor

All authors- Danika Jurat, Parveen Kumar & Kim Goddard.

## Provenance and peer review

Not commissioned, externally peer-reviewed.
